# Novel cannula design improves large volume auto-injection rates for high viscosity solutions

**DOI:** 10.1080/10717544.2021.2018069

**Published:** 2021-12-28

**Authors:** Bruce C. Roberts, Christopher Rini, Rick Klug, Douglas B. Sherman, Didier Morel, Ronald J. Pettis

**Affiliations:** a Translational and Clinical Sciences Center of Excellence, BD Technologies and Innovation, Research Triangle Park, NC, USA; b BD Clinical Development, Pont-de-Claix, France

**Keywords:** Large volume subcutaneous injection, autoinjector, pre-filled syringe, *in vivo*, high viscosity, ultra-thin wall cannula, injection depth, tissue response

## Abstract

A prototype reusable large-volume (2 mL) autoinjector (LVAI) was designed to compare injection performance of a novel 27 gauge ultra-thin wall (UTW) pre-filled syringe (PFS) cannula (8 mm external cannula length, 14.4 mm total needle length) against an existing 27 gauge special thin wall (STW) PFS cannula (12.7 mm external cannula length, 19 mm total needle length) across a range of injectate viscosities (2.3–30 cP) in a series of *in vivo* feasibility studies in swine. The UTW cannula had an approximately 30% greater cross-sectional lumen area than the STW cannula. The target exposed needle length was adjusted to ensure appropriate needle penetration depth and achieve injectate deposition in the subcutaneous (SC) tissue. Delivery time and volume, injection site leakage, injectate depot location, and local tissue effects were examined. The STW and UTW cannulae both provided effective SC delivery of contrast placebo solutions, and were able to accommodate injectate viscosity up to 30 cP without quantifiable leakage from the tissue and with minor tissue effects which resolved within 1–2 hours. Delivery times at each viscosity were significantly different between PFS types with the UTW PFS producing faster delivery times. In a histological substudy of the UTW cannula using injectate viscosities up to 50 cP, injection site reactions were rare and, when present, were of minimal severity. This series of studies demonstrates the feasibility of LVAI SC injection and informs autoinjector and PFS design considerations. Use of a UTW cannula may enable more rapid LVAI injections with minimal tissue effects, especially for higher viscosity formulations.

## Introduction

In recent decades, there has been rapid growth in biologic therapies for diverse chronic conditions including diabetes, rheumatoid arthritis, cancer, autoimmune diseases, and multiple sclerosis, among others (Jones, 2017; Bittner, 2018; Hu, 2020; Abolhassani et al., 2012). Biologic therapies are typically administered parenterally through intravenous (IV) or subcutaneous (SC) routes because effective oral delivery methods are not yet available (Wright, 2020). Subcutaneous injection using pre-filled syringes (PFSs) and/or autoinjectors (AIs) (Hu, 2020) is a well-established and effective route for drug administration and provides several advantages over IV delivery. Such devices enable self-administration by patients or at-home caretakers (Hudry, 2017; Ferguson et al., 2019; Bernstein, 2020; Frias, 2020), improve the convenience of therapy, and reduce time requirements and overall healthcare system costs (Rule, 2014; Jackisch, 2015; Olofsson, 2016; Jones, 2017).

Manufacturers of SC biotherapy devices must account for several key considerations. Many biologics are injected at high dosages to achieve their intended effect (Shire, 2004; Mathaes, 2016), which poses fewer challenges for IV infusion but may require larger volumes than are typical for the SC route. An industry threshold of ≤2 mL was long considered to be the maximum limit for SC administration, and most approved SC therapies are administered in volumes ≤1.5 mL (Mathaes, 2016). However, this injection volume threshold has been challenged in recent years, with numerous therapies now being developed for SC injection at higher volumes (Shapiro, 2010; Wasserman et al., 2011; Mathaes, 2016; Jones, 2017; Frias, 2020). While concentrating drug formulations may reduce dose volumes, this strategy may lead to more viscous solutions and require additional excipients for drug stabilization (Shire, 2004), and may still result in volumes above those considered typical for SC injection. Higher viscosity injectates are associated with greater SC tissue pressures and increased injection pressure forces and delivery durations for the injection systems (Allmendinger, 2015; Doughty, 2016; Jones, 2017) and increased hold times may be needed to minimize leakage from the injection site (Doughty, 2016). These longer delivery times may in turn impede user acceptance for required hold duration. Such factors must all be considered during SC injection device design.

SC AIs are well-established with a history of effective use and have recently been adapted for delivery of volumes up to 2 mL (Mathaes, 2016; Jones, 2017). Given the growing demand for SC biologics across many therapeutic areas, there is a need for AIs capable of delivering these larger volumes (>1 mL) with higher viscosity solutions while reducing injection pressures and minimizing local tissue effects. As injection forces of concentrated solutions are impacted by cannula properties (Allmendinger, 2014), needles with larger internal diameter may improve the flow dynamics of high viscosity liquids and shorten injection durations for patients. With this in mind, a prototype reusable 2 mL AI was designed to compare injection performance of a novel 27-gauge ultra-thin wall (UTW) PFS cannula (8 mm external cannula length, 14.4 mm total needle length) against an existing 27-gauge special thin wall (STW) PFS cannula (12.7 mm external cannula length, 19 mm total needle length) across a range of injectate viscosities (2.3–30 cP) in a series of *in vivo* animal feasibility studies. PFS exposed needle length beyond the AI face was examined and adjusted to ensure appropriate needle penetration depth (NPD) and to achieve target injectate deposition in the SC tissue. Additional key parameters that were evaluated included delivery time and volume, injection site leakage, injectate depot locations in tissue, and local tissue effects over time, including a histological evaluation of tissue effects following UTW injections using viscosities up to 50 cP.

## Materials and methods

### Large-volume autoinjector test devices and PFS configurations

**Figure 1.**
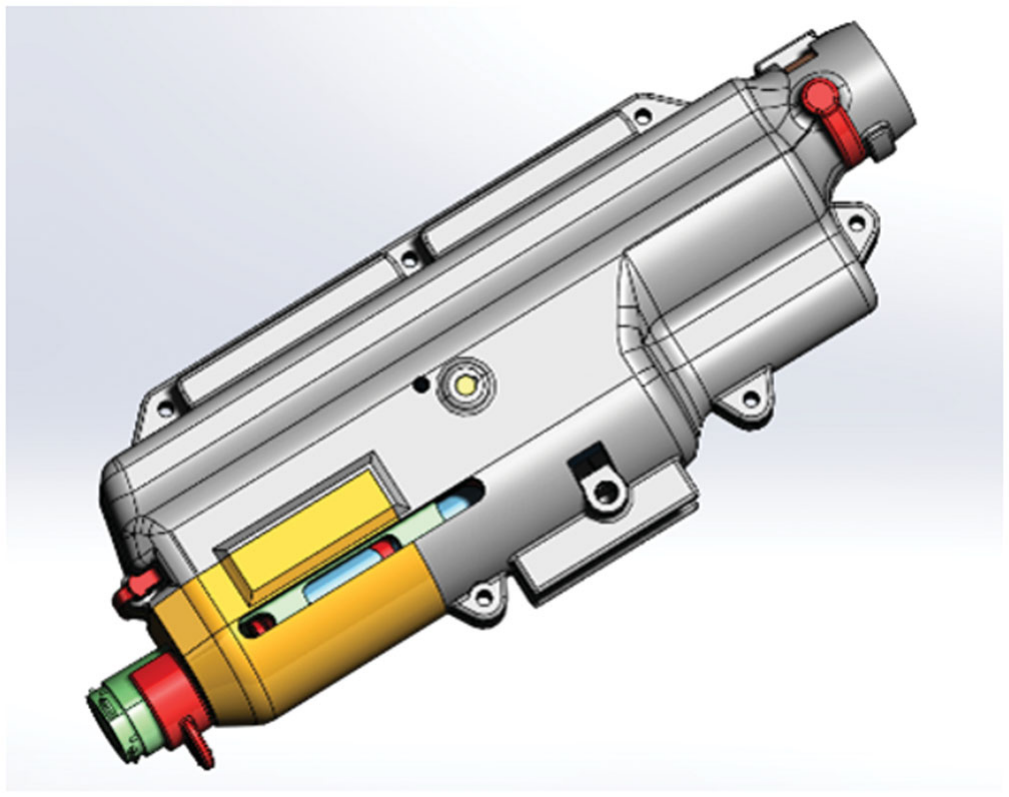
Reusable large-volume 2 mL autoinjector prototype. The prototype device design provided a flexible platform to assess delivery performance due to its ability to interchange drive springs, select needle extension length, and replace PFS components post-delivery.

A reusable large-volume AI (LVAI) prototype device (BD, Research Triangle Park, NC) was designed with adjustable features to allow examination of different PFS cannula types, exposed needle lengths (i.e. needle extension beyond the LVAI body) and drive spring forces (Figure 1). A combination of additive manufacturing processes including 3D printing, stereolithography, direct metal laser sintering, and machined metal parts were used to fabricate reusable prototype components. The LVAI utilized a drive spring that produced 46.7 ± 1.9 N force when compressed (start position of the spring before AI activation) and 28.2 ± 2.2 N force at 1.36 inches of expansion/travel (spring extended length after AI injection), hereafter referred to as 50 N springs.

LVAI delivery performance was assessed for two 2.25 mL PFS configurations: a commercially available 27-gauge STW needle (BD Neopak™, Pont-de-Claix, France), and a proprietary prototype 27-gauge UTW needle with an approximately 30% greater cross-sectional lumen area, each with FluroTec^®^ stoppers. A replaceable depth limiter was used to adjust the exposed needle length beyond the AI face.

### Benchtop assessments

Benchtop delivery characterization (delivery duration, delivered volume, and exposed needle length) of reusable prototypes with STW PFS components was conducted across 2.3, 20, and 30 cP solution viscosities to examine delivery performance against atmosphere prior to *in vivo* testing. Delivery start and end times were identified from accumulated weight versus time profile utilizing a custom MATLAB script. Delivery volume was measured gravimetrically using a calibrated balance with real time data acquisition and calculated based on solution density (delivery volume (mL)= mass/placebo density). Exposed needle lengths post AI activation were measured under ×60 magnification.

### Solutions for injection

Newtonian placebos were commercial iohexol contrast solutions with nominal viscosities of 2.3 cP, 10 cP, and 20 cP at 20 °C (Omnipaque™ 140 mg, 300 mg, and 350 mg iodine/mL, respectively; GE Healthcare, Oslo, Norway). Additional higher viscosity placebo solutions were custom formulated at 30 cP, 40 cP, and 50 cP from iohexol contrast (reagent grade, Sigma-Aldrich Histodenz^TM^, St. Louis, MO) with dextran (Dextran 40 MW 40,000, pharmaceutical grade, Pharmacosmos, Holbaek, Denmark) added as a viscosity modifier. Placebo viscosities were confirmed on a Brookfield LVDV III + rheometer (AMATEK Brookfield, Middleborough, MA) at 20 °C using a CPE-40 spindle. Shear rate scans were performed manually for each solution between 10% and 100% full scale range. Injection placebo densities were measured on a DMA 4500M densitometer (Anton Paar, Graz, Austria) at 20.00 °C, 21.50 °C, and 23.00 °C. Pre-fillable syringes were manually filled with 2.0 mL of the target solution using a positive displacement pipette followed by vacuum stoppering.

### Animal model

For *in vivo* studies, the reusable prototype LVAI device was tested on 30–40 kg anesthetized female Yorkshire swine. Swine are a preferred tissue injection model for preclinical evaluation of injection devices, having key similarities to human dermal and SC tissues (Swindle, 2012, 2015). The flank intradermal (ID) and SC tissue thickness provide a representative analog to human thigh and abdomen injection sites with sufficient surface area for multiple 2 mL injections without significant discomfort or physical impairment to the animals.

AI injections were administered to flanks of anesthetized swine in a standardized grid pattern with six injection sites per side from anterior to posterior for a maximum of 12 injection sites per animal per injection period. Injection order and grid location were randomized by device and balanced across individual animals. Prototype AI injections used push-on-skin activation applied perpendicular to the skin surface without skin pinch-up.

All animal care and preclinical procedures were conducted in accordance with National Research Council Standards of Care and Ethics at an Association for Assessment and Accreditation of Laboratory Animal Care accredited facility under an experimental protocol approved by the responsible Institutional Animal Care and Use Committee.

### NPD and tissue effects as a function of AI exposed needle length

An *in vivo* study was completed using the STW PFS to assess target versus actual *in vivo* NPD, and to evaluate the impact of exposed needle length on injection site effects. Results were used to select the preferred exposed needle length for subsequent contrast injection studies. Injections of 2.0 mL of 0.9% normal saline without contrast media enabled visualization of the cannula tip position and tissue penetration depth by 2D fluoroscopic images (Glenbrook Technologies Labscope™, Randolph, NJ) taken perpendicular to the injection. A total of 105 injections were administered at three target post-activation exposed needle lengths: 4 mm (*n* = 34), 6 mm (*n* = 36), and 8 mm (*n* = 35). Fluoroscopic images and NPD measurements were taken during each insertion and injection, and actual exposed needle lengths were also measured post-activation. The tissue NPDs were used as inputs for an ID and intramuscular (IM) *in silico* statistical risk model. Injection-induced erythema (redness) and edema (wheal) were each recorded immediately following injection (0 = none; 1 = slight, 2 = well defined, 3 = moderate, and 4 = severe) using a grading scale from the Organisation for Economic Co-operation and Development Guideline for the Testing of Chemicals (OECD, 2015) as done in other studies using a porcine model (Kang, 2013). For this study, wheal and redness responses are assumed to be induced by the injection process rather than by an immunological response.

### *In silico* injection risk modeling

The risks of ID and IM injection were calculated using an *in silico* probability model which uses empirical NPD measurements of published average human tissue thickness (Gibney, 2010; Hirsch, 2014) at four common SC injection sites (abdomen, thigh, arm, and buttocks) in adults, pooled and stratified by factor (sex, body mass index (BMI)) in accordance with previously published methods (Rini, 2019). Tissue ID thickness, tissue IM depth, and device NPD values were generated using a random number generator function from the respective distributions. Monte Carlo simulations were run at a sample size of *n* = 500 for each combination of tissue thickness and NPD. For each pair of (a) ID depth and NPD, and (b) IM depth and NPD, the ID and IM risk was calculated as the proportion of tests where either NPD < ID depth or NPD > IM depth, respectively.

### Evaluation of delivery time and volume, injectate leakage, depot location, and tissue effects using *in vivo* contrast injections

Based on ID/IM risk modeling results from the saline injection study, a 6 mm exposed needle length was selected for further evaluation. Large volume (2.0 mL) injections of contrast agent (*n* = 163) across three viscosities (2.3, 20, and 30 cP) were administered to the flanks of anesthetized Yorkshire swine in the same manner as in the NPD study using the reusable prototype AI with 50 N drive spring and two PFS cannula types (STW: *n* = 60 and UTW: *n* = 103) at a 6 mm target exposed needle length.

Injection delivery time was captured with a stopwatch while visually monitoring syringe stopper travel. Delivered volume was determined by differential gravimetric analysis using pre- and post-injection PFS weights and injectate solution density. Once delivery was complete or 30 seconds following activation (whichever occurred first), the device was held in place for five seconds and then removed from the tissue. Any injectate leakage from the tissue was captured using absorbent sponges and quantified by gravimetric analysis (Laurent, 2006). Leakage amounts too small to reliably quantify (<10 µL), were recorded in a separate category.

Calibrated axial measurements from the skin surface to the closest (top) and furthest (bottom) depot border were obtained for each 2.0 mL delivery using a 2D fluoroscope (Glenbrook Technologies Labscope™, Randolph, NJ).

### Fluoroscope-based measurements

Measurements made with fluoroscopic imagery were calibrated against a radiopaque scale (1.0 mm gradations) placed in the field of view during image capture. A parallax correction factor was applied to all measurements to accommodate the minor magnification difference between the position of the imaged tissue and the calibration standard relative to the X-ray source. The correction factor was consistent for all measurements and applied to the data prior to statistical analysis.

Injection site erythema (redness) and edema (wheal) effects were assessed as in the NPD study immediately after injection and at hourly intervals up to four hours; although, exact hourly time points differed between the STW and UTW studies. If site effects did not resolve within four hours post-injection, a final observation was completed at 24 hours. If all injection sites on an animal resolved to a score of 0 for both erythema and edema, no further observations were completed.

### Evaluation of histopathological tissue response for UTW cannula injections

In an additional substudy, tissue samples were collected to evaluate localized injection-related tissue response using the reusable AI prototype and 27-gauge UTW PFS administering 2 mL of contrast agent across a 2.3–50 cP viscosity range (*n* = 3 injections per each of six injectate viscosities: 2.3, 10, 20, 30, 40, and 50 cP) in six female Yorkshire swine (30–40 kg) as per prior injections. Skin punch biopsies (10 mm in diameter) were collected at 0-, 2-, and 24-hours post-injection (*n* = 3 per time point per viscosity); biopsies of naïve controls were also collected. Biopsies were bisected longitudinally and fixed in 10% buffered formalin. Using routine processing for paraffin-embedded sections, slides of each section were stained with hematoxylin and eosin for light microscopy. Local tissue response for each sample was scored individually for the epidermis, dermis, and subcutis by a board-certified veterinary pathologist on a five-point scale (0 = none, 1 = minimal, 2 = mild, 3 = moderate, and 4 = severe). These scores were averaged to provide a composite irritation score based on the guidelines established by the American National Standards Institute (ANSI) and Association for the Advancement of Medical Instrumentation (AAMI).

### Statistical analysis

Needle penetration depth endpoints were analyzed using linear models, and erythema/edema scores using cumulative logit models, with device and viscosity factors and their interaction as explanatory variables. Multiple comparisons were performed per device and per device per viscosity using contrast methods when the models underlying assumptions were met.

Paired Student’s *t*-tests were conducted, without multiple comparison adjustment, to assess differences between measured NPD and target exposed needle length (4, 6, or 8 mm). For the analysis of delivery time and volume, when assumptions underlying linear regression models were not met, the Wilcoxon two-way ANOVA for medians test (as implemented in R package WRS2) was used to test the significance of device type, viscosity and their interaction, and multiple comparisons were performed using Wilcoxon’s tests adjusted for multiple comparisons with Sidak's method. A *p* value of .05 was used as the significance level.

## Results

### Benchtop characterization shows consistent performance of the LVAI device

The AI prototypes used for subsequent *in vivo* injections demonstrated consistent performance in benchtop assessments: the exposed needle lengths were measured within +0.85 mm/–0.3 mm of their target lengths (4, 6, and 8 mm) post-activation, the delivered volume was within ±5% of 2.0 mL, and the variation in delivery time across viscosities was low (<1.05 seconds).

### 6 mm as a preferred exposed needle length

The results of the *in silico* risk modeling analysis were as expected, with the shallower injections having a higher ID injection risk and deeper injection depths having a higher IM injection risk (Table 1). As IM risk is directly related to SC tissue thickness, the thigh presented the highest calculated risk, followed by arm, abdomen, and buttock injection sites.

**Table 1.** Mean NPD and calculated ID and IM injection risk per device configuration at thigh and abdomen injection sites, pooled across age, sex, and BMI.

Device configuration	NPD Mean ± SD	Calculated ID risk	Calculated IM risk
STW 4 mm	4.46 ± 0.53	Thigh (0.0%), abdomen (0.0%)	Thigh (3.1%), abdomen (0.4%)
STW 6 mm	6.48 ± 0.38	Thigh (0.0%), abdomen (0.0%)	Thigh (13.4%), abdomen (3.1%)
STW 8 mm	8.83 ± 0.48	Thigh (0.0%), abdomen (0.0%)	Thigh (32.5%), abdomen (12.4%)

BMI: body mass index; ID: intradermal; IM: intramuscular; NPD: needle penetration depth; SD: standard deviation; STW: special thin wall.

**Figure 2.**
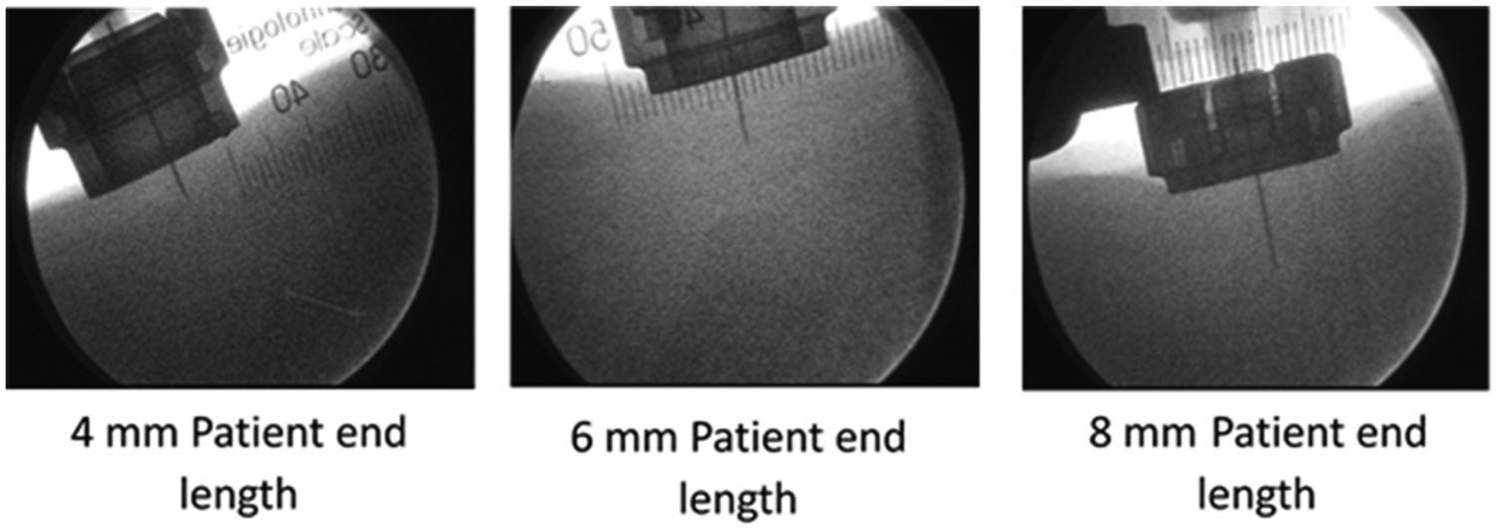
Fluoroscopic image of the needle in tissue during injection; the device interface with the skin surface and cannula are clearly visualized allowing measurements of needle penetration depth.

For the saline-only injections using the STW PFS (Figure 2), *in vivo* NPD measurements (mean ± standard deviation (SD)) demonstrated a statistically significant (*p*<.001) positive bias from the target depth (4 mm: 4.46 ± 0.53 mm; 6 mm: 6.48 ± 0.38 mm; 8 mm: 8.83 ± 0.48 mm) for each configuration. However, actual post-activation exposed needle length measurements (mean ± SD) from the AI face to the needle tip were also slightly higher than expected across target NPDs (4 mm: 4.83 ± 0.21 mm; 6 mm: 6.81 ± 0.32 mm; 8 mm: 9.02 ± 0.14 mm).

**Figure 3.**
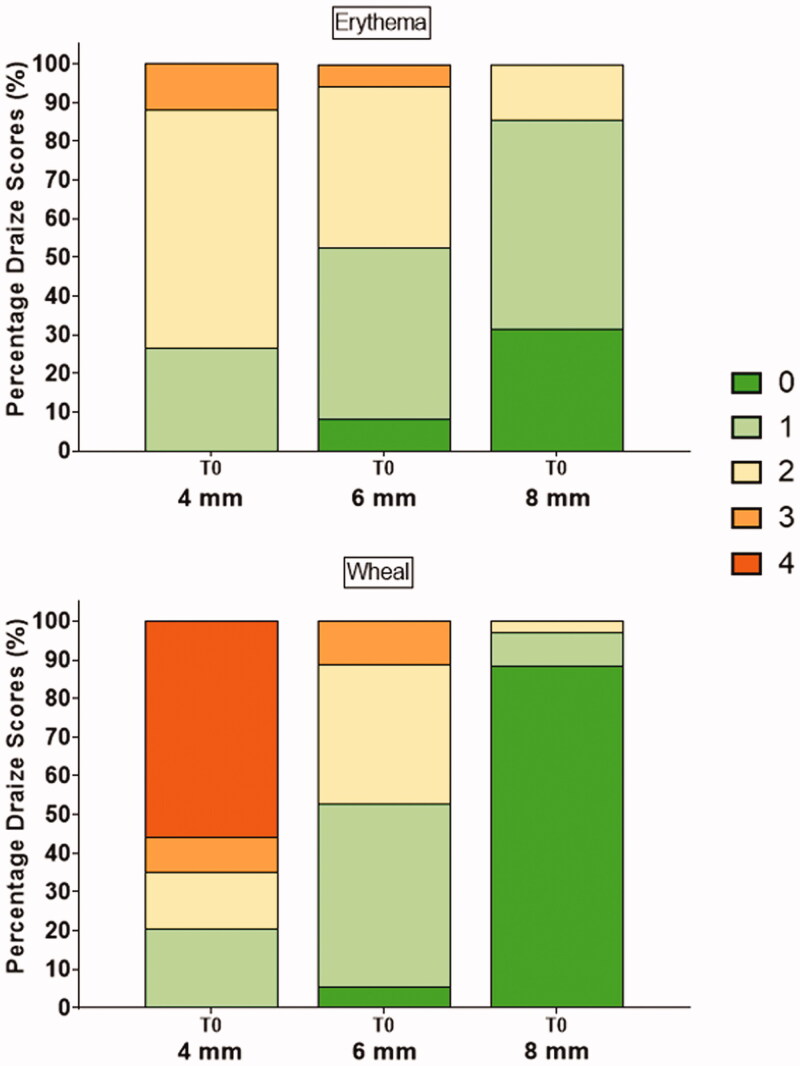
Observed erythema and wheal scores (0–4) by target exposed needle lengths of 4, 6, and 8 mm immediately after 2 mL saline injection. Shorter exposed needle length was associated with higher scores.

Statistically significant (*p*<.001) increased erythema and edema scores immediately after injection were associated with shorter exposed needle lengths (Figure 3); the most pronounced injection site effects were observed when using the ∼4 mm exposed needle length. Although injection site effects were reduced significantly with the 8 mm target needle length, this also increased the IM risk potential calculated using the *in silico* model. To balance between these two factors, the 6 mm exposed needle length was used for subsequent preclinical evaluations of 2.0 mL SC AI injections.

### Contrast injections demonstrate successful 2.0 mL SC delivery for high viscosity injectates using both STW and UTW cannulae

Of the 163 contrast injections administered, 155 injections were available for analysis (STW, *n* = 52; UTW, *n* = 103). For the STW configuration, *n* = 2 injections were excluded due to weighing errors, and *n* = 6 injections were excluded due to prototype AI activation or component failures. For the UTW configuration, there were no exclusions.

**Figure 4.**
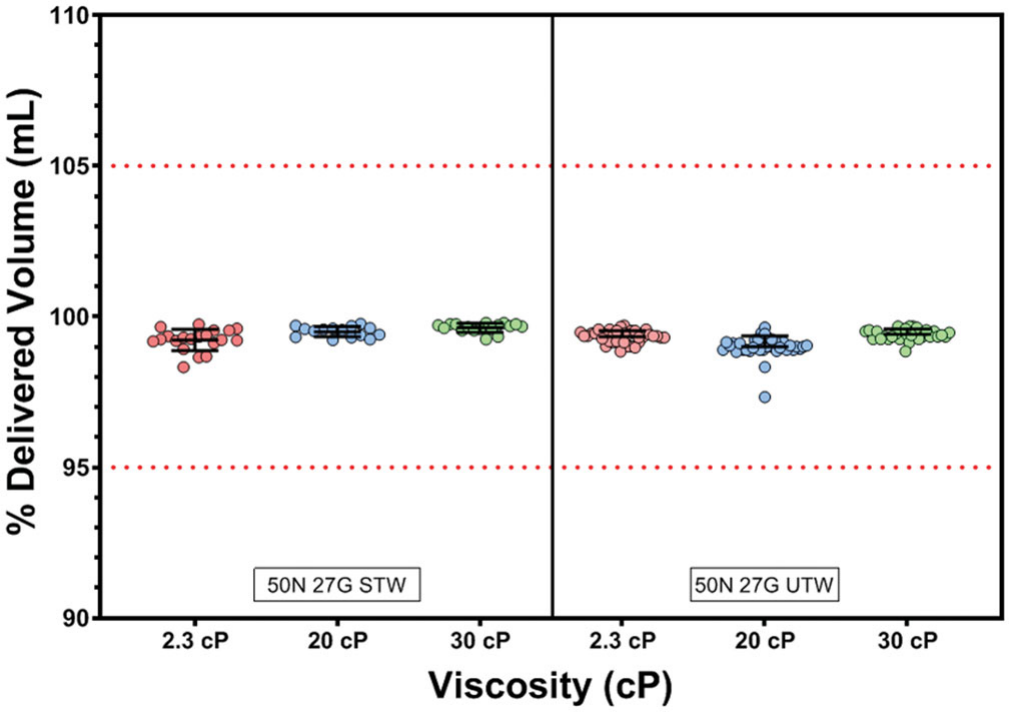
Percent delivered volume by PFS and viscosity. Delivered volumes for each PFS/viscosity combination were within ±5% of filled syringe volume.

**Figure 5.**
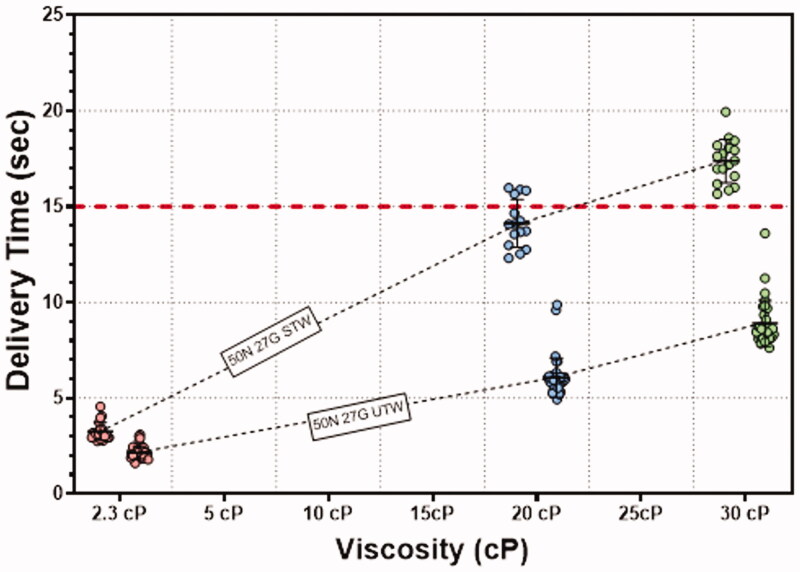
LVAI 2 mL delivery time (seconds) by PFS and viscosity. As expected, delivery time increased with increasing viscosity, and significant differences in delivery time were observed between viscosity levels. Mean delivery times were less than 15 seconds at all tested viscosity levels using the UTW PFS, and at 2.3 and 20 cP using the STW PFS. The UTW PFS produced a significant reduction in delivery time at 20 and 30 cP compared to the STW PFS.

All LVAI delivered volumes were within ±5% of the filled syringe volume (Figure 4) for both the STW and UTW PFS configurations. ANOVA analysis results indicated a significant effect of device and viscosity on delivery volume (*p*<.001). Subsequent multiple comparison tests were performed and demonstrated a statistically significant difference between PFS configurations at 20 cP and 30 cP. Despite their statistical significance, these differences were not considered of practical or clinical significance as all delivered volume measurements were within the identified ±5% target range of 1.90 mL to 2.10 mL. Leakage was rare for all PFS/viscosity combinations and, when visible, was not quantifiable by differential gravimetric analysis (<10 µL). As expected, ANOVA analysis confirmed viscosity and PFS configuration as significant factors for delivery time (*p*<.001). Mean delivery times were less than 15 seconds for the majority of test PFS/viscosity configurations, with the exception of the STW/30 cP test group at 17.4 seconds. Delivery times at each viscosity were significantly different between PFS types with the UTW PFS producing faster delivery times (Figure 5, Table 2; *p*<.001).

**Table 2.** Mean delivery times for *in vivo* deliveries by mean injectate viscosity.

PFS	STW			UTW		
Viscosity	2.3 cP	20 cP	30 cP	2.3 cP	20 cP	30 cP
Mean (s)	3.26	14.13	17.37	2.15	6.07	8.9
SD	0.48	1.25	1.13	0.34	1.02	1.21
*n*	20	15	17	36	36	31

PFS: pre-filled syringe; SD: standard deviation; STW: standard thin wall; UTW: ultra-thin wall.

### Injectate deposition was within the target SC tissue

**Figure 6.**
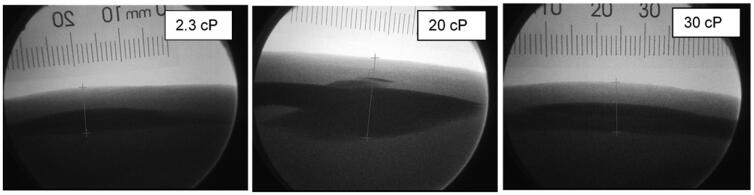
Representative fluoroscopic images of 2 mL depositions in tissue by viscosity. The surface of the skin is readily evident due to its stark contrast with the air surrounding the animal under the fluoroscope. The injectate depositions appear as dark areas in the image with distinct top and bottom borders. All injectate placebos used for deposition imaging in this study had sufficient radiological density to be readily visible by fluoroscopy. The interface between the dermal and subcutaneous space is also evident in the images, typically 2–3 mm from the surface of the skin.

**Figure 7.**
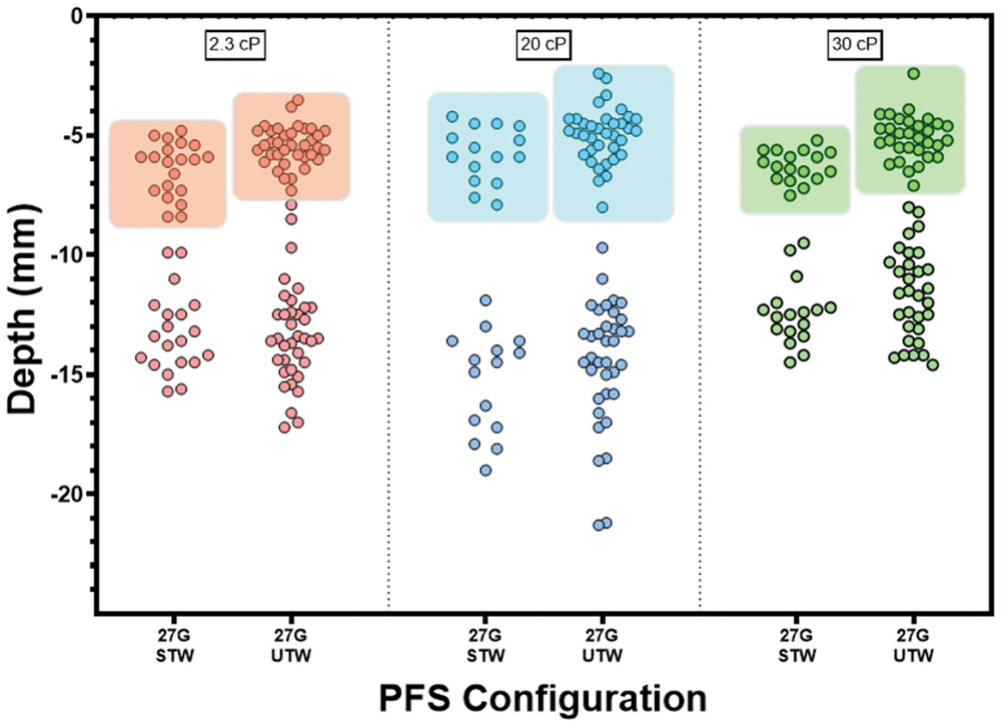
Depot top (within colored boxes) and bottom border measurements (mean ± SD) per PFS and viscosity. All deliveries were confirmed within the SC tissue via fluoroscopic imaging. The 0.0 *y*-axis represents the skin surface.

Fluoroscopic images of LVAI 2 mL contrast agent delivery showed that all depositions resided in the target SC tissue space (Figure 6). Between PFS configurations and within viscosity, the STW PFS top of depot measurements was statistically significantly deeper than the UTW PFS (Table 3, Figure 7; 20 cP, *p*=.006; 2.3 cP and 30 cP, *p*<.001 for both). This difference is likely attributed to the slightly longer exposed needle length from the 6 mm target as measured in the NPD study (STW: 6.81 ± 0.32 mm; UTW: 6.02 ± 0.30 mm). No statistically significant differences for depth measurements to the bottom of the injectate depositions were observed between PFS configurations.

**Table 3.** Descriptive statistics for depth measurements to the top and bottom of the deposition from the surface of the skin by PFS and injectate viscosity.

PFS	27-gauge STW			27-gauge UTW		
Viscosity	2.3 cP	20 cP	30 cP	2.3 cP	20 cP	30 cP
*Top border deposition*						
Mean (mm)	6.38	5.80	6.26	5.43	4.97	5.07
SD	1.13	1.16	0.64	0.81	1.14	0.92
*n*	20	15	17	36	36	31
*Bottom border deposition*						
Mean (mm)	13.27	15.29	12.44	13.34	14.47	11.63
SD	1.68	2.12	1.36	2.09	2.58	1.89
*n*	20	15	17	36	36	31

PFS: pre-filled syringe; SD: standard deviation; STW: standard thin wall; UTW: ultra-thin wall.

### Injection site erythema was lower with the UTW than with the STW cannula

**Figure 8.**
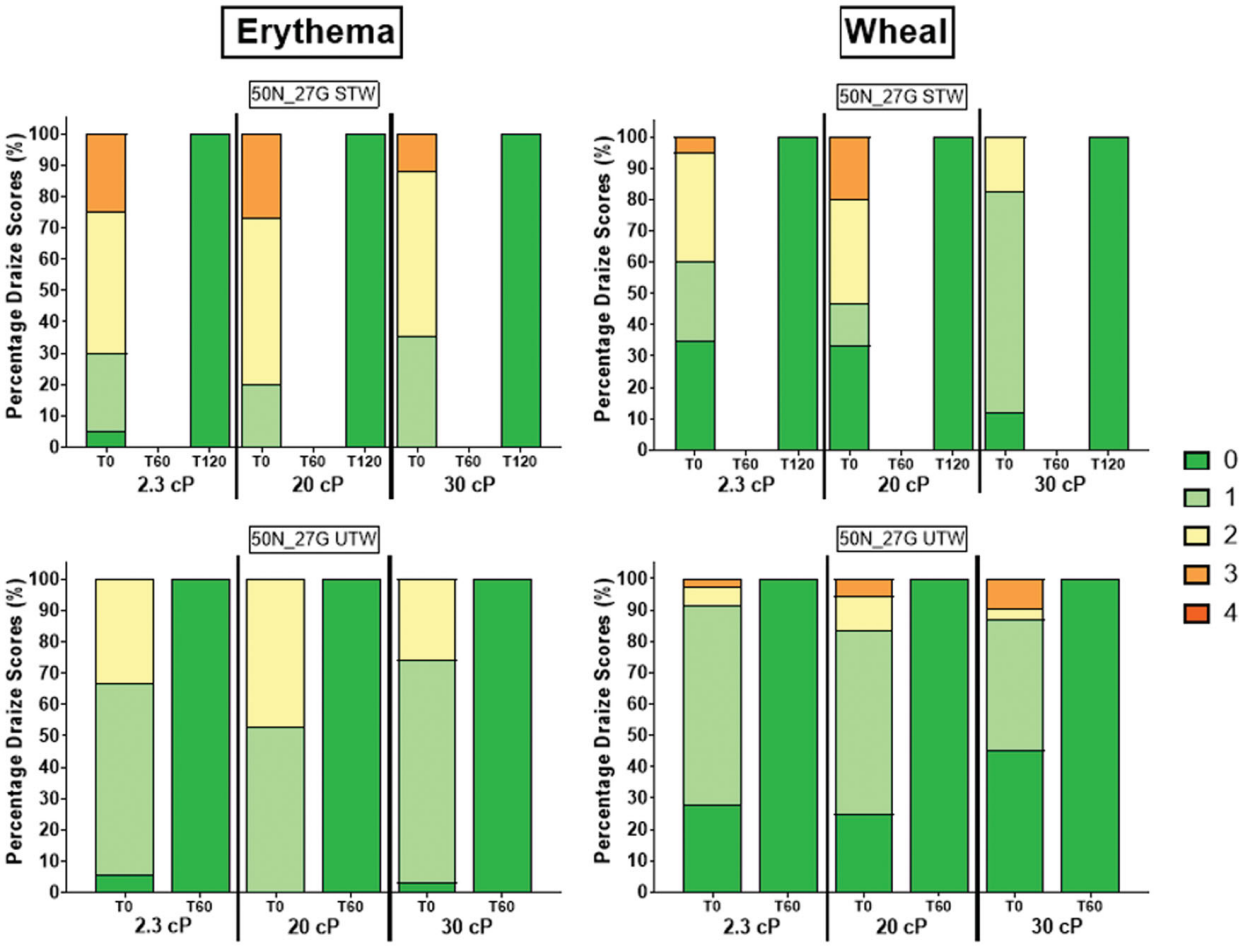
Percentage of observed erythema (left) and edema (right) scores (0–4) for tested device configurations immediately after injection and at T-60 minutes and T-120 minutes, stratified by viscosity. T-120 minute observations for the UTW PFS deliveries were unnecessary as complete resolution occurred by T-60 minutes. Injection site effect resolution occurred within 1–2 hours post 2 mL injections. T-60 observations were not made for STW cannula injections.

Percentages of injection sites in each erythema/edema score category (0–4) immediately following injection (*T* = 0 minutes) and at post-delivery time-points (*T* = 60 minutes, *T* = 120 minutes) are displayed in Figure 8. The majority of post-injection site effect scores were modest (score ≤2) across PFS configurations and viscosity levels, with complete resolution occurring within two hours. Irritation score differences were observed between PFS types, showing higher erythema scores with STW use across all viscosities (2.3 cP: *p*= <.001; 20 cP: *p*=.004; 30 cP: *p*=.006). Non-significant edema score differences were observed between PFS types within viscosity levels.

### UTW cannula injections had minimal tissue response in histological analysis

In general, tissue reactions were rare and limited to focal, serocellular crusts with hyperkeratosis of the epidermis or minimal hemorrhage and edema deposition within the SC tissues. Average irritant scores at two hours post-injection were less than those observed at 24 hours. Inflammatory changes such as polymorphonuclear (neutrophilic) infiltrates, fibrin, edema, necrosis, or hemorrhage were rare and of minimal severity when present. Average irritant scores at all time points never exceeded 1.8 (*T* = 24 hours: ≤1.8, *T* = 2 hours: ≤0.5, and *T* = 0 hours: ≤0.8) which does not exceed the threshold for designation as an irritant (2.9) as defined by the guidelines established by the ANSI and AAMI (non-irritant = 0–2.9, slight irritant = 3.0–8.9, moderate irritant=(9.0–15.0, severe irritant >15). Naïve controls irritant scores did not exceed 0.3, with an average score 0.06 across all naïve sites.

## Discussion

In this series of early phase feasibility studies, a reusable prototype LVAI was developed and used to successfully deliver 2 mL SC injections of varying viscosities with both STW and UTW PFS configurations. Both cannulae provided effective large-volume SC delivery and were able to accommodate injectate viscosity up to 30 cP without quantifiable leakage and with only nominal observations of skin surface droplets (∼10 µL) and minor tissue effects which resolved quickly over time.

A key difference in overall cannula performance was the significantly lower mean injection time observed for UTW cannula deliveries compared to those using the standard STW cannula, particularly at higher viscosities. This was not unexpected since flow varies quadratically with diameter and linearly with length based on typical Hagen–Poiseuille fluid dynamics principles. This benefit of a more rapid delivery rate was not abrogated by *in vivo* tissue flow resistance and suggests that the UTW cannula may reduce clinical delivery durations under otherwise identical injection conditions. The data demonstrate high viscosity delivery performance advantages with the use of an increased cannula lumen cross sectional area, and these advantages are achievable without increasing the cannula’s outside diameter. While there is a theoretical lower limit for cannula wall thickness both for manufacturability and to avoid affecting mechanical integrity and impacting delivery success, that limit is not currently known; cannula integrity was maintained with no mechanical failures observed during the conduct of this study. AIs have shown high usability for self-administration of SC therapy in various patient populations, including older adults and patients with limitations associated with rheumatoid arthritis (Hudry, 2017; Xiao et al., 2018; Ferguson et al., 2019; Bernstein, 2020; Frias, 2020). The decreased UTW cannula delivery time may allow for shorter LVAI hold times, which could benefit patient usability. Additionally, the UTW cannula may expand the applicable formulation viscosity ranges for LVAI injections. Thus, use of the UTW cannula may represent a key design element for effective LVAI delivery rate control and optimization.

Injection site effects for both PFS configurations were minor post-injection with complete resolution within 1–2 hours. UTW cannula injections showed less erythema immediately after injection compared with the STW cannula, with a similar but non-significant trend observed for edema. Furthermore, in the histological UTW cannula substudy, injection site reactions were rare and, when present, were of minimal severity. There were no apparent differences in local tissue response based on contrast agent viscosity at any of the examined time points in the histological study. These data suggest that the more rapid UTW LVAI injection rates do not increase adverse tissue effects but would require further evaluation to determine if these nominal benefits translate clinically. UTW injection pain and perception cannot be determined in preclinical animal models, and would also benefit from additional clinical comparison studies in pertinent LVAI user populations.

Injection depth was a key study consideration to ensure that deposition was occurring in the target SC tissue space. As demonstrated by the injection data using a 4 mm target needle length, shallow injections, even when fully deposited within the SC tissue, may increase observed tissue effects and their resolution time. Conversely, deeper 8 mm injections may reduce visible surface tissue effects but increase the IM injection risk according to *in silico* model predictions. In the *in vivo* studies, both the STW and UTW PFS configurations resulted in injectate deposition within the SC tissue. However, the STW cannula produced slightly deeper depositions as measured from the skin surface to the depot top. This was most likely attributable to minor increases in actual exposed needle length as measured on post-injection study prototypes rather than any effect of between-cannula internal diameters. These results inform the need for both appropriate overall LVAI and PFS injection depth targets and effective overall system tolerances to achieve target SC injections while minimizing visible tissue effects that could impact user therapy acceptance. Again, further evaluation is warranted in a clinical setting, especially using specific therapies of interest which may also impact tissue outcomes.

This series of studies should be interpreted within the context of their potential limitations. The reusable prototype device utilized herein was developed without human factor optimization as a research tool to explore and inform the LVAI and PFS design and engineering space, and the device itself is not intended for subsequent development. Integrated LVAI usability and performance should be subsequently examined. A preclinical swine model was used for the evaluation of NPD and deposition location via fluoroscopy to avoid repeated human subject exposure to ionizing radiation during imaging. Swine are a well-accepted translational model for human skin and SC tissue. However, nominal tissue differences do exist, highlighting the need for subsequent clinical evaluation with a more patient-friendly imaging methodology. Delivery performance was characterized using bespoke Newtonian placebos including imaging contrast across specific viscosity levels, and these early feasibility studies could not evaluate the impact of prototype AI design nor formulation on patient perception. Prior work has shown that large volume, high viscosity injections are well tolerated, and that rapid injection time does not significantly impact patient perception (Berteau, 2015; Dias, 2015; Usach, 2019). Needle characteristics also impact patient perception, with shorter and thinner walled needles correlated with improved patient perception and comfort (Watt, 2019). The overall tolerability and acceptability of any given combination product is impacted by a broad combination of factors including the anatomical injection site, device characteristics, and drug formulation. Therefore, delivery parameters, injection site effects, patient perception, and device acceptability should be further assessed under expected clinical use conditions for specific drug formulations.

In conclusion, the LVAI reusable prototype successfully delivered the target 2 mL volume injectates *in vivo* using STW and UTW PFS configurations. The UTW cannula significantly reduced measured injection times compared to the STW cannula, with minor associated injection site effects that resolved quickly post-injection. This series of studies demonstrates the feasibility of larger volume SC injection and informs AI and PFS design considerations, enabling more rapid LVAI injections especially for higher viscosity formulations.

## Data Availability

Due to its proprietary nature, supporting data cannot be made openly available.
